# Mortality Causes in Free-Ranging Eurasian Brown Bears (*Ursus arctos arctos*) in Spain 1998–2018

**DOI:** 10.3390/ani10091538

**Published:** 2020-08-31

**Authors:** Ana Balseiro, Luis J. Royo, Elena Gayo, Ramón Balsera, Olga Alarcia, Juan F. García Marín

**Affiliations:** 1 Departamento de Sanidad Animal, Facultad de Veterinaria, Universidad de León, 24071 León, Spain; elena.gayoroces@gmail.com (E.G.); jfgarm@unileon.es (J.F.G.M.); 2 Departamento de Sanidad Animal, Instituto de Ganadería de Montaña (CSIC-Universidad de León), Finca Marzanas, Grulleros, 24346 León, Spain; 3 Servicio Regional de Investigación y Desarrollo Agroalimentario del Principado de Asturias (SERIDA), 33300 Villaviciosa, Spain; ljroyo@serida.org; 4 Consejería de Fomento, Ordenación del Territorio y Medio Ambiente, Oviedo, 33007 Asturias, Spain; ramon.balserariesgo@asturias.org; 5 Consejería de Fomento y Medio Ambiente de la Junta de Castilla y León, Dirección General de Patrimonio Natural y Política Forestal, 47014 Valladolid, Spain; AlaAleOl@jcyl.es

**Keywords:** brown bear, *Ursus arctos arctos*, pathology, cause of death, infectious diseases, traumas

## Abstract

**Simple Summary:**

This work summarizes the mortality cases of twenty-five free-ranging Eurasian wild brown bears (*Ursus arctos arctos*) from the Cantabrian mountain range submitted for necropsy in Asturias and Castilla y León (northwestern Spain) from 1998 to 2018. Mortality cases were classified both caused by (i) “non-human intervention” or “human intervention” causes and based on (ii) “non-infectious” or “infectious” etiology. Based on “non-human intervention” or “human intervention”, fourteen of the 21 (66.7%) brown bears in which the cause of death could be determined died as a consequence of “non-human intervention” and seven (33.3%) by “human intervention”. Based on “non-infectious” or “infectious” etiology, twelve of the 21 (57.1%) brown bears died due to “non-infectious” etiology and the remaining nine (42.9%) animals due to “infectious” diseases. In a free-ranging population of Eurasian brown bear from the Cantabrian mountain range, main causes of death are attributed to non-human related traumatic lesions and infectious diseases (primary developed such as infectious canine hepatitis or secondary developed such as clostridiosis or septicemia).

**Abstract:**

This work summarizes the mortality cases of twenty-five free-ranging Eurasian wild brown bears (*Ursus arctos arctos*) from the Cantabrian mountain range submitted for necropsy in Asturias and Castilla y León (northwestern Spain) from 1998 to 2018. Mortality cases were classified both caused by (i) “non-human intervention” or “human intervention” causes and based on (ii) “non-infectious” or “infectious” etiology. In four cases (16%) it was not possible to determine the cause of death due to the inadequate preservation of collected specimens or insufficient tissue availability. Based on “non-human intervention” or “human intervention” causes, fourteen of the 21 (66.7%) brown bears died as a consequence of “non-human intervention” due to traumatic lesions (fights, unknown traumas or infanticide), infectious canine hepatitis, neoplasia or mushroom poisoning. In contrast, seven (33.3%) brown bears died by “human intervention” due to illegal hunting (shooting or snare), handling (during transit in an attempt to reintroduce a bear back into the wild) or strychnine poisoning. Based on “non-infectious” or “infectious” etiology, twelve of the 21 (57.1%) brown bears died due to “non-infectious” causes, namely traumatic lesions such as shooting, snare, fighting or infanticide, handling, strychnine poisoning, mushroom poisoning or neoplasia. The remaining nine (42.9%) animals died due to “infectious” diseases which included gangrenous myositis, infectious canine hepatitis or septicemia. In six of those cases traumatic lesions caused by non-human or human activities were complicated with bacterial infection (clostridiosis and septicemia) which finally caused the death of those animals. Additionally, exertional myopathy was observed in the handled animal and in one bear found in a snare. In a free-ranging population of Eurasian brown bear from the Cantabrian mountain range, main causes of death are attributed to non-human related traumatic lesions and infectious diseases (primary developed such as infectious canine hepatitis or secondary developed such as clostridiosis or septicemia) which is in contrast to previously reported data for other bear populations. These data are valuable and may help in the conservation and management of this recovering population.

## 1. Introduction

In Spain, the endangered Eurasian brown bear (*Ursus arctos arctos*) population is located in the Cantabrian mountain range (northwestern Iberian Peninsula) and represents the southwestern limit distribution for this species in Europe [1]. The population of free-ranging Eurasian brown bear has recovered during the last two decades (2000–2020) from approximately 100 individuals in the 1990s to 230–260 presently [1,2]. Brown bears in the Cantabrian range are divided into two subpopulations (western and eastern) separated by about 50 km, almost isolated from a genetic point of view for over a century and with little connection between them [1,2]. The western population has approximately 200 individuals and can be found in Galicia, Asturias and Castilla y León. The eastern population includes about 30 individuals and occupies a small area in Asturias, Palencia (eastern León) and Cantabria [2]. On an international level, the Cantabrian brown bear is listed on the International Union for Conservation of Nature (IUCN) Red List of Threatened Species and cataloged as in danger of extinction [3].

The identification of causes of mortality in wild natural populations is relevant for the correct design of conservation strategies and management programs [4]. However, knowledge on the mortality among bears is limited because of the difficulty in finding the dead animals in nature, and certain percentage of dead animals remains unrecorded.

The deaths of brown bears in nature sometimes can be attributed to human interventions, i.e., shooting, poisoning or traffic accidents, and on other occasions death is due to natural causes, i.e., infanticide [4,5].

The aim of the present work is to summarize confirmed causes of deaths and most significant findings related to deaths reported in twenty-five free-ranging Eurasian brown bears submitted for necropsy in Asturias and Castilla y León (northwestern Spain) over the past 20 years. These data are valuable and may help in the conservation and management of this recovering population.

## 2. Materials and Methods

### 2.1. Study Area

The bear’s usual area of distribution in the Cantabrian range is about 7700 square kilometers (approximately from 42°53′ N; 7°10′ W to 42°53′ N; 4°02′ W) [2,3]. It is characterized by an Atlantic climate with a temperature range from −4 to 8 °C in the coldest months and moderate precipitation throughout the year (900–1400 mm per year) [6]. It gives suitable habitat to brown bears and includes mountain woodlands, abrupt relief, forests and dense vegetation cover that provides refuge and food throughout the year.

### 2.2. Data Collection

Twenty-five free-ranging Eurasian brown bears—16 from Asturias and 9 from Castilla y León, of different sexes (16 males and 6 females) and ages (20 subadult/adults and 5 cubs) were necropsied from 1998 to 2018. Sex could not be determined for three animals.

After detecting each dead animal and the thorough investigation of the area (e.g., signs of fight) where the bears were found, they were preserved at 4 °C and a complete post mortem examination of each carcass was conducted at the University of León or Servicio Regional de Investigación y Desarrollo Agroalimentario (SERIDA, Asturias) in less than 24 h. We complied with the IUCN Policy Statement on Research Involving Species at Risk of Extinction and the Convention on the Trade in Endangered Species of Wild Fauna and Flora. We also complied with the national and regional legislation on sampling of Cantabrian brown bear (Consejería de Fomento, Government of Principality of Asturias and Consejería de Fomento y Medio Ambiente, Junta de Castilla y León). Ethics approval was deemed unnecessary according to Spanish national regulations (Real Decreto 53/2013).

### 2.3. Diagnostic Procedures

After necropsy tissue samples (encephalon, spinal cord, tongue, heart and skeletal muscle, lungs, liver, gallbladder, kidneys, adrenal glands, urinary bladder, spleen, pancreas, gut, genital tract and lymph nodes) were taken for evaluation using standard methods in histology [7], microbiology [7], molecular techniques [8], virology [8], parasitology (coprologic study), toxicology and histopathology [7,8,9]. Histological stains included hematoxylin–eosin, Gram, Ziehl–Neelsen, Von-Kossa, Mallory–Azan, Klüber–Barrera and Masson’s trichrome. A dental histological study of the first molar or upper canine tooth [10] was performed when possible in order to determine the age of the bears. Data on bears carcass sites, necropsy findings and laboratory results were considered in making conclusions about the cause of death and allowed tracking of changes and trends in mortality throughout the years.

## 3. Results

### 3.1. Classification of Causes of Death

**Figure 1:**
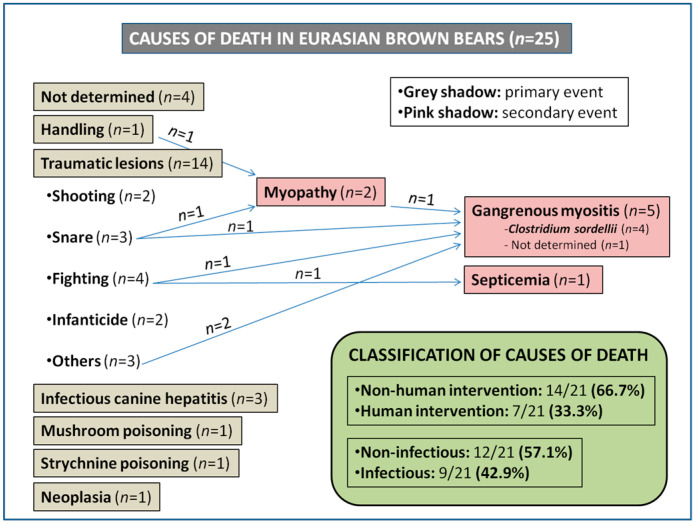
Mortality cases in Eurasian brown bears (*Ursus arctos arctos*) in Asturias and Castilla y León (northwestern Spain) from 1998 to 2018. Blue arrows indicate bears which died from a combination of human activities or non-human related trauma and secondary bacterial infection.

The cause of death and relevant pathologic findings in the twenty-five brown bears studied is shown in Table 1 and Figure 1. In four (4/25, 16%) cases it was not possible to determine the cause of death due to inadequate preservation of collected specimens or insufficient tissue availability. Three causes of death (*Clostridium sordellii* infection in one bear, infectious canine hepatitis caused by canine adenovirus type 1 (CAdV-1) in three bears and cholangiocarcinoma in one bear) were already described in previous papers by our group [7,8,9].

Mortality cases were classified both caused by (i) “non-human intervention” or “human intervention” causes and based on (ii) “non-infectious” or “infectious” etiology. Based on caused by “non-human intervention” or “human intervention”, fourteen of the 21 (14/21, 66.7%) brown bears in which the cause of death could be determined died as a consequence of “non-human intervention” due to traumatic lesions (*n* = 9, 42.9%) [fights (*n* = 4, 19%), unknown traumas (*n* = 3, 14.3%) or infanticide (*n* = 2, 9.5%)], infectious canine hepatitis (*n* = 3, 14.3%), neoplasia (*n* = 1, 4.8%) or mushroom poisoning (*n* = 1, 4.8%). In contrast, seven (7/21, 33.3%) brown bears died by “human intervention” due to illegal hunting [wire snare hunting (*n* = 3, 14.3%) and shooting (*n* = 2, 9.5%)], handling (*n* = 1, 4.8%) and strychnine poisoning (*n* = 1, 4.8%). Mortality data were also stratified based on “non-infectious” or “infectious” etiology. Twelve of the 21 (12/21, 57.1%) brown bears died due to “non-infectious” causes, namely traumatic lesions (*n* = 8, 38.1%) such as shooting, snare, fighting or infanticide, handling (*n* = 1, 4.8%), strychnine poisoning (*n* = 1, 4.8%), mushroom poisoning (*n* = 1, 4.8%) or neoplasia (*n* = 1, 4.8%). The remaining nine (9/21, 42.9%) animals died due to “infectious” diseases, which included gangrenous myositis (*n* = 5, 23.8%; four of which were demonstrated to be from clostridiosis), infectious canine hepatitis (*n* = 3, 14.3%) or septicemia (*n* = 1, 4.8%). As shown in Figure 1, the pathologic findings on six animals revealed secondary bacterial infection followed by primary traumatic event caused either by human activities (i.e., wire snare hunting) or non-human related causes (i.e., fights or unknown traumas). Exertional myopathy was observed in two bears, a handled animal and a bear found in a snare.

### 3.2. Description of Causes of Death

Cases of death caused by traumas such as shooting (metallic fragments were found), wire snares, fighting or infanticide were easier to determine not only based on necropsy and histopathological studies, but also using complementary diagnostic techniques (i.e., radiography) or knowledge of the behavior of this species. Ante mortem traumatic lesions always showed vascular reactions such as hemorrhages, hyperemia, congestion or edema. In fighting and infanticide cases lesions in skin, muscles or bones showed signs compatible with bites and scratches. In two animals the origin of traumas could not be determined. Other causes were more difficult to discern and are reported below.

### 3.3. Infectious Diseases

The five bears (Bears 1, 8, 10, 15 and 23) with secondary gangrenous myositis (Figure 1, Table 1), as a consequence of wire snare hunting, infanticide, fights or traumas, showed serohemorrhagic edema in the abdominal cavity, thorax, pericardium and skeletal muscle and hemorrhages in heart, skeletal muscles, stomach, intestine, liver, spleen and kidney. The case description of Bear 8 was previously reported [7]. Microscopically, vascular damage and hyperacute myodegeneration consisted of myonecrosis, edema, gas, extravasation of fibrin into the interstitial spaces, and lacunar dissolution of myofibers in skeletal muscles were observed using hematoxylin–eosin and Masson´s trichrome stains in those animals. *Clostridium sordellii* was identified by Gram stain and culture [7] as the etiological agent of the lesions in four brown bears. In two of these four bears each, the *C. bifermentans* and *C. septicum* were also isolated. The presence of *C. sordellii* was always associated with previous muscle damage (i.e., traumas) that triggered its proliferation. In Bear 15 *Clostridium* spp. could not be isolated. An additional bear (number 12) showed septicemia secondary to fighting although the etiological agent could not be determined.

Gross lesions in the three bears (Bears 11, 14 and 20) with infectious canine hepatitis caused by CAdV-1 were previously described [8] and consisted of congestion and hemorrhages in several organs; hemorrhagic fluid in thoracic and abdominal cavities; friable and yellow-tinged liver; hepatomegaly; and thickening of the gall bladder due to edema. Microscopically the main pathologic findings appeared in liver and gall bladder [8]. The liver showed mild centrilobular multifocal degeneration and necrosis of hepatocytes, with the presence of intranuclear inclusions bodies and low inflammatory infiltration mainly of lymphocytes. The gall bladder showed edema of the wall. Additionally, non-purulent encephalitis was observed. CAdV-1 was confirmed by quantitative polymerase chain reaction (qPCR) and immunohistochemistry [8].

### 3.4. Exertional (Degenerative) Myopathy

**Figure 2:**
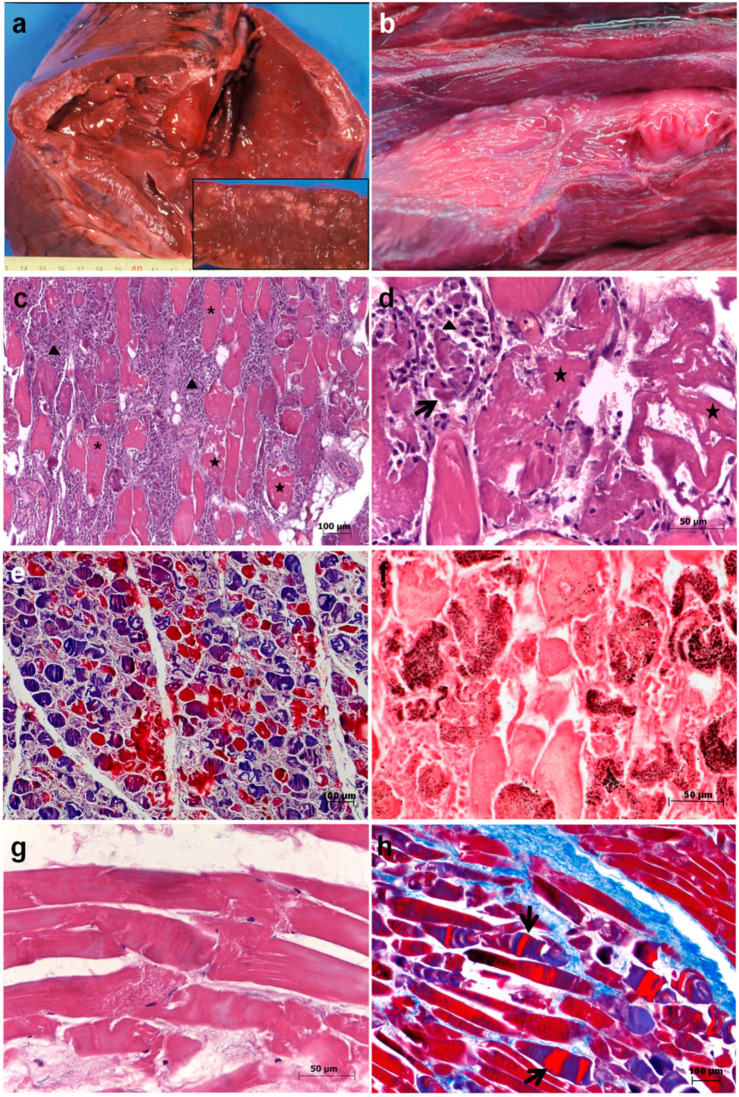
Severe exertional myopathy in Eurasian brown bears. (**a**,**c**,**d**,**e**,**f**) Bear 1; (**b**,**g**,**h**) Bear 9 (see Table 1 for details). (**a**) Gross lesions consisted of dry and pale cardiac muscle. Inset: detail of lesions in myocardium; (**b**) gross lesions consisted of pale skeletal (femoral) muscle; (**c**) extensive segmental hyaline degeneration in femoral muscle, hypercontracted fibers (asterisks), coagulative necrosis and areas of myofibrillar lysis (stars), as well as intensive infiltrate mainly consisted of lymphocytes and macrophages (arrowheads), hematoxylin–eosin staining; (**d**) detail of necrotic myofibers (stars) with surviving satellite cells (arrow), invading macrophages (arrowhead) and elongating myoblasts indicative of events of regeneration, hematoxylin–eosin staining; (**e**) necrotic and lysed myofibers, Mallory–Azan staining; (**f**) calcification (mineralization), hyalinization and necrosis of muscle fibers, Von Kossa staining; (**g**) segmental degeneration in a longitudinal section of intercostal muscle. In this case infiltrate is not present, hematoxylin–eosin staining; (**h**) necrotic and hypercontracted (arrows) myofibers, Mallory–Azan staining.

In two animals, exertional myopathy was diagnosed. One bear (Bear 1) additionally exhibited signs of clostridiosis after being captured in a snare and died after one week. A female cub also died due to exertional myopathy after handling (Bear 9). The cub was found alone in the wild when it was two months old and it was kept in captivity until it was nine months old. The cub died during transit in an attempt to reintroduce it back into the wild. Both animals showed gross lesions consisting of dry and pale cardiac and some skeletal (mainly intercostals and femoral) muscles (Figure 2a,b). Microscopically, severe segmental degeneration of muscles was observed and consisted of hypercontracted fibers, extensive Zenker´s hyaline degeneration and coagulative necrosis of myofibers (Figure 2c–h). Bear 1 also showed an intensive infiltrate mainly consisting of lymphocytes and macrophages, as well as mineralization in the affected muscles (Figure 2c,f). In that animal necrotic myofibers with surviving satellite cells, invading macrophages and elongating myoblasts, all indicative of events of regeneration, were also observed (Figure 2d). The cub also showed hypoplasia of adrenal glands (1.7 g, 0.004% relative weight; physiological relative weight 0.03% [11]).

### 3.5. Strychnine Poisoning

In the bear affected by strychnine poisoning (Bear 6) extensive hemorrhages were found in several organs (heart, lungs, liver, kidney, spleen, stomach and intestine) also showing hemothorax, hemopericardium and hemoperitoneum. Microscopically vascular damage and diffuse necrosis in those organs were the most common findings. Strychnine was identified by chromatography from hair samples in a private company.

### 3.6. Neoplasia

Cholangiocarcinoma was observed in the liver of an old female (Bear 13). A complete description of this case was already published [9]. Briefly, microscopically liver tumor tissue showed tubular, acinar or pseudoglandular structures in the area facing a large cavity of necrosis with a thick trabecular growth pattern. Multiple small nodules were also present in the gall bladder. Metastatic encapsulated foci of cholangiocarcinoma were located in lung parenchyma, adrenal glands and articulation of the left elbow.

### 3.7. Mushroom Poisoning

Bear 19 showed hemorrhagic gastritis and diffuse hepatic and renal necrosis compatible with mushroom poisoning, likely due to ingestion of poisonous *Amanita* spp., although that could not be confirmed by toxicological analysis. Other poisons/toxics/toxins were not detected.

## 4. Discussion

This study shows that the main causes of death in Eurasian brown bears from the Cantabrian mountain range are those caused by non-human related traumas and infectious diseases, mainly by clostridiosis (associated with previous muscle damage) or infectious canine hepatitis. As an example, in Sweden, where ninety-eight animals were analyzed, no deaths of brown bears were attributed to any infectious disease [5]. Thus, the confirmation of *C. sordellii* secondary to traumas and CAdV-1 (the agent of infectious canine hepatitis in dogs) as relevant causes of death in Eurasian brown bear is probably the most outstanding finding in this study. In contrast with previous data on brown bears, for the first time infectious diseases are described as an important cause of death. The four fatal cases of clostridial infection described to date, compared to the few descriptions reported worldwide, could show a higher susceptibility of the Cantabrian brown bear population to these bacteria [7]. Gross lesions such as hemorrhages and edema in skeletal muscle and internal organs may lead to suspect of clostridial infection in bears found dead in the nature. The putative high susceptibility to infectious pathogens reported here may be a consequence of a weaker immune system due to the lower genetic diversity described for this species in the Cantabrian range subpopulation [12,13]. Another possibility may be a high degree of circulation of the pathogens both in the environment and sympatric species (i.e., CAdV-1 carriers such as wolves or not sanitarily controlled dogs in rural areas) [8].

Two animals died showing exertional myopathy as a consequence of extreme exertion and stress after trapping by a snare [14] or handling. Those are to our knowledge the second and third cases of death by exertional myopathy reported in bears in the literature worldwide. The first case was described by Cattet et al. (2008) in a grizzly bear that died approximately 10 days after capture by leghold snare in Canada [15]. Those three cases confirm the fact that the prolonged suffering in bears may cause exertional myopathy as occurs in other mammal species, i.e., red fox or cervids [16,17]. Therefore, that should be taken into account in the management of bears, especially when capturing and handling free-living specimens, trying to minimize stress as much as possible. Some measures to reduce stress and complications due to capture myopathy may include minimize noise and activity, limit captures to periods with reduced temperature or provide sodium bicarbonate, Selenium or Vitamin E supplements [17]. The acute presentation of adrenal hypoplasia shown in the cub could be precipitated by the physiologic stress suffered [18].

The detection of four male bears that died after fighting (fight events were just observed in males), three due to traumas, three due to CAdV-1 infection, two cubs due to infanticide, one due to a cholangiocarcinoma and one due to mushroom poisoning confirm the existence of deaths attributable to “non-human-mediated” natural causes usually difficult to detect in nature, and thus highlight the importance of necropsies.

Nevertheless, the confirmation of six of 25 brown bears submitted to the laboratory for necropsy as death by shooting, snare and poisoning (four from 1998 to 2008 and two from 2008 onwards) seems to agree with the apparent reduction in illegal killing after the mid-1990s [1,19], suggested as a key factor for the recovery undergone during last two decades [2], which would represent an example of successful coexistence between human and bears in the Cantabrian range. In this regard, society education is crucial for the species conservation.

The causes of death should be clearly differentiated in small and isolated populations like in Spain, Italy or France with causes of death in large and hunted populations like in Canada. Similar to our study, in the Trentino region of Italy [20] and in the French Pyrenees [21] which are also small and isolated populations, the main cause of death is from natural causes. In contrary, in large and hunted populations the main cause of death is human-mediated. In this regard human actions were responsible of most grizzly bear mortalities in Canada [4]. In Sweden, Mörner et al. (2005) found that human caused mortality stands for 64% of total submissions; meanwhile bears killed by other bears and infanticide were the most frequent natural cause of death (16%) [5]. Reljic et al. (2018) and Krofel et al. (2012) reported for hunted populations in Croatia and Slovenia, respectively, more than 90% human caused mortality, mostly hunting, traffic collisions and intervention removals [22,23].

Traffic induced bear mortality has been documented as a common cause of death in bears in other European countries [5,22,23,24,25,26,27,28,29]. In our study, we have not reported any road-killed animals, despite one bear that died in a traffic accident (the only one reported in Spain to date) in León in 2008, although necropsy of this bear was not performed, and it is not included in this report [30].

Despite the limited number of studied cases (related to the conservation status and behavior of the species that hampers the detection of moribund or dead bears in nature) the data reported in the present work confirm the persistence and relevance of causes of death directly related to human intervention. The total number of mortalities and causes of death is a conservative figure as unreported mortality is difficult to estimate. In this sense small carcasses from cubs or yearlings are even more difficult to find, therefore this study may be also limited due to the biased sampling. Additionally, the impossibility of establishing the cause of death in four brown bears submitted for necropsy due to the nature and/or preservation status of available samples must be taken into account when considering the frequency and importance of the different causes of death reported in this study.

Regarding the limited genetic variability of the Cantabrian brown bear population [2]—and taking into account that a free-ranging bear can live about 25 years [2,19]—we can consider that the three animals found dead at the age of 19–20 years likely had the opportunity to widely contribute to the genetic stock of the Cantabrian brown bear population. However, two of the five necropsied cubs (40%) died due to CAdV-1 infection. Thus, we can consider that an important percentage of future progenitors may die due to this infectious disease among others causes, which can be important for the maintenance of genetic variability of the species—even more in this endangered population.

## 5. Conclusions

In summary, the data in the present work represent a first review of the causes of death confirmed at necropsy in the Cantabrian brown bear population since 1998. In this free-ranging population of Eurasian brown bear from the Cantabrian mountain range, the main causes of death were attributed to non-human related causes, mostly due to traumatic lesions and infectious diseases (primary developed such as infectious canine hepatitis or secondary developed such as clostridiosis or septicemia), which is in contrast to previously reported data for other bear populations. The apparent moderate, but steady recovery experienced during last decades provides a new landscape for this endangered population, where recovery and conservation works will must be accompanied by an increasingly important effort on surveillance and management programs of the species, focused on the control of infectious diseases shared between domestic and wild animals under the one health strategy.

## Figures and Tables

**Table 1:** Available data, cause of death, pathologic findings and classification of death of twenty-five free-ranging Eurasian brown bears (*Ursus arctos arctos*) necropsied from 1998 to 2018 in Asturias and Castilla y León (northwestern Spain).

Bear	Date	Age	Sex	Cause of Death	Classification of Death
1	8/May/1998	7 years	Male	Snare/exertional myopathy/gangrenous myositis (*Clostridium sordellii* and *C. bifermentans*)	H/I
2	12/June/1998	Cub	Female	Infanticide	NH/NI
3	10/June/2000	Adult	n.d.	n.d.	–
4	6/June/2005	Subadult	Male	n.d.	–
5	26/September/2005	Adult	Male	Shooting	H/NI
6	19/November/2005	Adult	n.d.	Poisoning: strychnine	H/NI
7	14/June/2008	Cub (1 year)	Male	Infanticide	NH/NI
8	27/August/2012	Adult	Male	Snare/gangrenous myositis (*Clostridium sordellii*) *	H/I
9	29/October/2012	Cub (9 months)	Female	Died after handling and transport/exertional myopathy	H/NI
10	12/June/2014	3 years	Male	Fighting/gangrenous myositis (*Clostridium sordellii* and *C. septicum*)	NH/I
11	15/June/2014	5 years	Male	Infectious disease: CAdV-1 **	NH/I
12	12/December/2014	9 years	Male	Fighting/septicemia	NH/I
13	29/April/2015	20 years	Female	Neoplasia: cholangiocarcinoma ***	NH/NI
14	23/May/2015	Cub (4 months)	Male	Infectious disease: CAdV-1 **	NH/I
15	16/October/2015	Adult	Male	Traumatic lesions/gangrenous myositis	NH/I
16	5/March/2016	Adult	Male	Traumatic lesions due to fall	NH/NI
17	8/October/2016	Subadult	Male	Shooting	H/NI
18	27/November/2016	6 years	Female	Snare/strangled	H/NI
19	7/January/2017	6 years	Male	Mushroom poisoning; hepatic an renal necrosis	NH/NI
20	3/April/2017	Cub (3 months)	Female	Infectious disease: CAdV-1 **	NH/I
21	21/April/2017	19 years	Male	Fighting and cliff fall	NH/NI
22	21/April/2017	20 years	Male	Fighting and cliff fall	NH/NI
23	29/September/2018	4 years	Female	Traumatic lesions/gangrenous myositis (*Clostridium sordellii*)	NH/I
24	27/October/2018	5 years	n.d.	n.d.	–
25	08/November/2018	7 years	Male	n.d.	–

* [7]. ** [8]. *** [9]. n.d.—not determined; CAdV-1—canine adenovirus type 1; H—human-intervention; NH—non-human intervention; I—infectious; NI—non-infectious; Bears 5, 7, 9, 11, 12, 15, 18, 24 and 25 are from Castilla y León. The remaining bears are from Asturias.
